# Some reminiscences on studies of age-dependent and activity-dependent degeneration of sensory and motor endings in mammalian skeletal muscle

**DOI:** 10.1111/joa.12334

**Published:** 2015-07-14

**Authors:** Richard R Ribchester

**Affiliations:** Euan MacDonald Centre for Motor Neurone Disease Research and Centre for Integrative Physiology, University of EdinburghGeorge Square, Edinburgh, UK

**Keywords:** muscle spindle, neuromuscular junction, neuroprotection

## Abstract

I present here an overview of research on the biology of neuromuscular sensory and motor endings that was inspired and influenced partly by my educational experience in the Department of Zoology at the University of Durham, from 1971 to 1974. I allude briefly to neuromuscular synaptic structure and function in dystrophic mice, influences of activity on synapse elimination in development and regeneration, and activity-dependent protection and degeneration of neuromuscular junctions in *Wld*^*S*^ mice.

It was extremely gratifying to return to Durham, at the invitation of Bob Banks and Guy Bewick, to present at this celebratory Symposium some of the research that has occupied me since I left Durham University in the mid-1970s, where I had been an undergraduate (BSc with Joint Honours in Chemistry and Zoology, 1974).

## Early days

I was profoundly influenced by my teachers in the Zoology Department, especially David Barker, and by his colleagues Ken Bowler, David Hyde, Mike Stacey, David Harker and Alice Milburn. In particular, we learned in our lectures and laboratory practical classes all about the controversial debate that prevailed during the late 1960s and throughout the 1970s (largely at odds with IA Boyd and his colleagues in Glasgow) over the question of segregation of the gamma motor innervation of the intrafusal nuclear bag and nuclear chain muscle fibres of muscle spindles in the cat, illustrated eloquently but dispassionately by some of the elegant experiments co-ordinated by Barker, that attempted to resolve this issue (Barker et al. 1977; Boyd et al. 1977). Barker’s lectures were among the most exciting, for me, of my time at Durham because they were patiently delivered with a great deal of thought, in a wholly engaging way, and founded on a balanced view of the evidence, including accounts and arguments based on ongoing and unpublished research. At the same time, I learned all about the ultrastructure of motor neurons, axons and neuromuscular junctions (NMJs) under Mike Stacey’s tutelage at the helm of the department’s electron microscope, and about mechanisms of neuromuscular transmission under David Hyde’s calm and insightful tutorial supervisions. From David Harker and Alice Milburn, I learned how to stain intramuscular nerves, using the silver chloride method so adroitly applied by the Barker lab, and to stain NMJs for cholinesterase activity, then to make teased preparations of this stained neuromuscular material for conventional light microscopy. I also carried out an undergraduate research project, on oxidative phosphorylation in blowfly flight muscle mitochondria, encouraged by the infectious enthusiasm and rigorous direction of Ken Bowler. Indeed, were it not for the lure of the bright lights (literally) of electrophysiological apparatus at the Muscular Dystrophy Laboratories in Newcastle, and a fortuitous series of circumstances that led me there, I would have stayed on with Bowler for my PhD training.

## Neuromuscular synaptic structure and function in dystrophic mice

As it turned out, however, I migrated a few miles up the road and took up post as an MRC PhD student at the Muscular Dystrophy Laboratories, under John B Harris’ expert supervision, in the autumn of 1974. However, I renewed my connections with Durham shortly afterwards. By that time, Barker’s group had been joined by Bob Banks, who brought and meticulously applied an approach that combined physiological recording and microanatomical description (Banks et al. 1978, 1998, 2009; Banks & Barker, 1989; Banks, 1994, 2006). Meanwhile, I honed the skills I had learned at Durham in single-fibre teasing, together with electrophysiological analysis of neuromuscular synaptic transmission that I learned from John Harris, and I subsequently brought them to bear on an experimental investigation that related to another controversy around that time, namely whether the signs and symptoms of muscular dystrophy were due to a significant amount of ‘functional denervation’ (Harris & Ribchester, 1979a). I also adapted a technique I first heard about during Barker’s lectures at Durham: intracellular staining of muscle fibres by microinjection of Procion dyes, in order to correlate microanatomy with electrophysiology (Barker et al. 1978). I put all these techniques and my acquired skills together, which enabled us to report with confidence that functional denervation was not a sufficient explanation for pathology in the signs and symptoms of murine muscular dystrophy in the *dy/dy* mouse mutant (Harris & Ribchester, 1978, 1979b).

## Neuromuscular synapse elimination in development and regeneration

An important lesson that I learned from Barker and his colleagues was to be ever vigilant and skeptical of tidy biological explanations: and that the prettiest explanation is not always the correct one. In the words of a guru from a quite different field of research (theoretical quantum electrodynamics), ‘the thing that doesn’t fit is the thing that is the most interesting’ (Feynman, 2001). This notion came to the fore again during my research a few years later, when – after postdoctoral fellowships abroad, mentored by Bill Betz in Denver (Betz et al. 1979, 1980a,b) and Jan Jansen in Oslo (Eide et al. 1982) – I was exploring the relationship between use and disuse (i.e. activity) of neuromuscular synapses during postnatal synapse elimination, a competitive process that occurs in rodents both during normal development (Brown et al. 1976; Betz et al. 1979, 1980a), and after nerve injury and regeneration in adults (McArdle, 1975; Ribchester & Taxt, 1983; Taxt, 1983). The prevailing view in the 1990s was that the outcome of synapse elimination was largely determined by differences in the activity of the axons converging and disposing their terminals at polyneuronally innervated NMJs (Ribchester & Taxt, 1983, 1984; Ridge & Betz, 1984; Ribchester, 1988; Betz et al. 1990; Balice-Gordon & Lichtman, 1994; Ribchester & Barry, 1994). However, I and my colleagues Jacqueline Barry and Ellen Costanzo showed that on the one hand, in reinnervated but experimentally paralysed muscle polyneuronal innervation may persist after activity has resumed; and conversely that synapse elimination and synaptic remodelling can still occur when muscles are completely paralysed, via a combined nerve conduction and neuromuscular transmission block. Together, these findings suggested that activity, though strongly influential on synapse elimination, is not decisive (Barry & Ribchester, 1995; Costanzo et al. 1999, 2000).

## Some intriguing aspects of neuromuscular synaptic protection in *Wld*^*S*^ mice

In the early-1990s, I began to turn my attention to the serendipitous discovery by Hugh Perry, Michael Brown and their colleagues of the mouse mutant now known as *Wld*^*S*^ (Lunn et al. 1989; Lyon et al. 1993; Coleman et al. 1998). In this strain, which turns out to have a tandem triplication of an 85-kb segment of genomic DNA, there is overexpression and cytoplasmic localization of a chimeric variant of the enzyme Nmnat-1. This enzyme catalyses synthesis of nicotinamide adenenine dinucleotide from its substrate nicotinamide mononucleotide (NMN). The chimeric Wld^S^ protein confers exceptionally strong protection on axons and their terminals from axotomy-induced Wallerian degeneration (Mack et al. 2001; Coleman & Freeman, 2010). The chimeric protein, which has a longer half-life than the axonal isoform Nmnat-2, substitutes for the precipitous loss of the latter isoform following axotomy (Gilley & Coleman, 2010; Conforti et al. 2014; Di Stefano et al. 2015).

**Fig 1:**
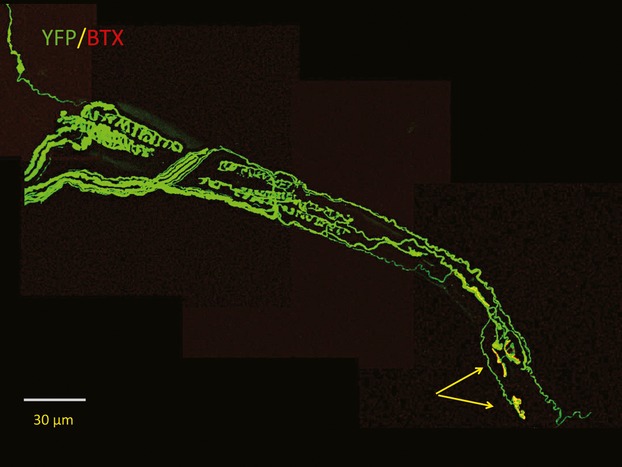
Confocal microscopic *z*-projection of annulospiral sensory and motor innervation (arrows) of a teased muscle spindle from transgenic mouse expressing Yellow Fluorescent Protein (YFP) in sensory and motor neurones (thy1.2YFP16; Feng et al. 2000; Oyebode et al. 2012). Acetylcholine receptors at the myoneural junctions were counterstained with a tetramethylrhodamine conjugate of alpha-bungarotoxin (BTX).

Neuromuscular sensory and motor axons and their terminals are especially well visualized in the F1 generation of *Wld*^*S*^ mice (which have no overt behavioural phenotype) cross-bred with transgenic mice expressing Yellow Fluorescent Protein (YFP) in sensory and motor neurons (Fig.1). We backcrossed these mice to make double homozygotes expressing the mutant *Wld*^*S*^ gene and the YFP transgene (Feng et al. 2000; Wong et al. 2009; Oyebode et al. 2012; Hirst & Ribchester, 2013). In the case of motor nerve terminals, onset of degeneration after axotomy in the double homozygotes, as in young (1–2 months old) homozygous *Wld*^*S*^ mice without the YFP transgene, is delayed by about 3 days. Degeneration occurs by progressive retraction of motor nerve terminals from motor endplates over the following 7 days, instead of the 12–24 h that is normally all that is required for complete degeneration in motor terminals in wild-type mice (Miledi & Slater, 1970; Winlow & Usherwood, 1975; Ribchester et al. 1995; Gillingwater et al. 2002; Bridge et al. 2009; Wong et al. 2009). Severed distal axons in *Wld*^*S*^ mice are protected for even longer: up to 3 weeks or more (Mack et al. 2001; Beirowski et al. 2005, 2009). Wld^S^ protein does not protect against cell death by apoptosis and, conversely, overexpression of anti-apoptotic genes does not protect axons from Wallerian degeneration (Sagot et al. 1995; Adalbert et al. 2006). This differential protection of neuronal compartments suggests that neuronal maintenance itself is compartmentalized, and that different but perhaps overlapping molecular processes regulate degeneration in somatic, axonal and terminal regions of a projection neurone (Gillingwater & Ribchester, 2001, 2003). This notion is supported by the discovery of novel point mutations, induced by ethylnitrosurea, that confer additive protection of axotomized neuromuscular synapses (Wong et al. 2009), although protection of axotomized motor terminals can also be extended by increasing the proportion of Wld^S^ protein that is retained in a motor neurone’s cytoplasm (Beirowski et al. 2009; Wong et al. 2009; Babetto et al. 2010). Recent studies have implicated both accumulation of NMN, the normal substrate for Nmnat-2, and Sterile Alpha And HEAT/Armadillo Motif Protein-1 (Sarm1) as upstream and downstream components of axons that may be toxic to them following nerve injury (Osterloh et al. 2012; Di Stefano et al. 2015).

**Fig 2:**
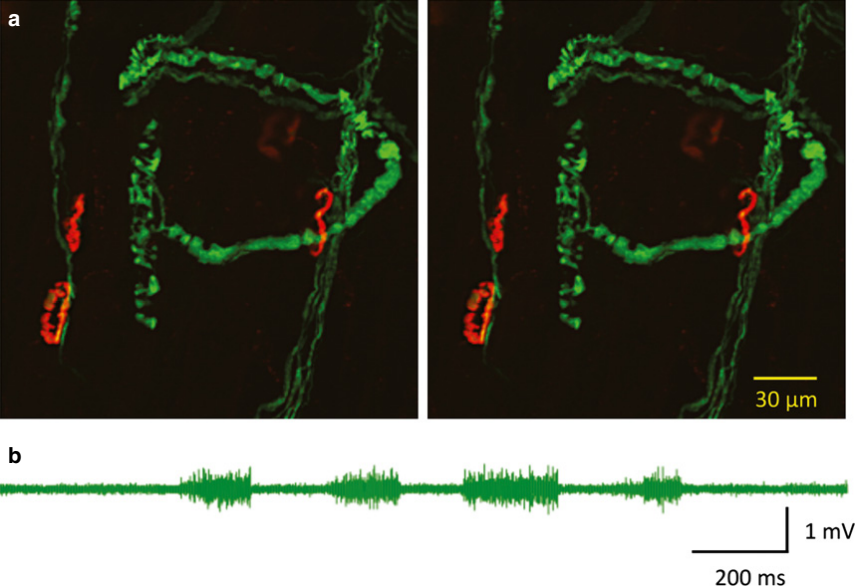
(a) Confocal microscopic *z*-projections at −6 ° and +6 °, through a transgenic/mutant *thy1.2YFP16/Wld*^*S*^ mouse lumbrical muscle, 5 days after sectioning the tibial nerve. Motor endplates were counterstained with TRITC-a-BTX. The two images may be viewed as a stereo pair using standard viewing methods. (b) Extracellular recording from the tibial nerve, 5 days after sciatic nerve section, during manipulation of the hindfoot and ankles. The sensory discharges indicate functional persistence of the axotomized distal sensory endings and axons residually innervating the limb.

Interestingly, the *Wld*^*S*^ phenotype is modifiable by several genetic, intrinsic and environmental determinants, most notably age, gene-copy number and the localization of the protective protein (Gillingwater et al. 2002; Beirowski et al. 2009; Wong et al. 2009; Babetto et al. 2010). However, another important phenotypic difference is observed in substantially greater protection of the annulospiral sensory axons and their endings on muscle spindles, compared with alpha motor axons and their terminals on extrafusal muscle fibres (Brown et al. 1994; Oyebode et al. 2012). The primary afferent endings of muscle spindles in hindfoot lumbrical muscles are especially well preserved up to 20 days after axotomy (Fig.2a), even in heterozygous *Wld*^*S*^ mice, or in aged mice, in which the protection of motor nerve terminals (but not axons) is lost within 24–48 h (Oyebode et al. 2012).

## Neuromuscular synaptic degeneration is sensitive to activity

The cause of the selective, enhanced protection of the Ia afferent endings compared with motor axons is unknown but could be related, at least partly, to differences in the level of expression or cytoplasmic localization of the Wld^S^ protective protein, as forced retention of this protein in the cytoplasm enhances protection (Beirowski et al. 2009; Babetto et al. 2010). However, an additional, notable and physiological difference between isolated sensory and motor endings in axotomized *Wld*^*S*^ mice is that axotomized sensory endings continue to respond, generating and propagating action potentials in the severed distal axons (Fig.2b), for as long as the sensory endings remain intact (Oyebode et al. 2012); whereas, of course, orthodromic activity in the case of motor neurons is propagated from cell body to axon terminal, and this pathway is broken when the axons are cut.

To test whether differences in activity might influence or determine the sensorimotor differences in rate of terminal degeneration, we recently conducted experiments utilizing isolated flexor digitorum brevis and lumbrical muscle preparations from *Wld*^*S*^ mice. We found that these preparations, in contrast to those from wild-type mice, survive and continue to function for at least 48 h when maintained in oxygenated physiological saline at a constant temperature of 32 °C (Brown et al. 2015). This allowed us to test for a modifying affect of activity. Thus, if activity were protective, this should extend the survival time of NMJ’s *ex vivo*. However, we found only liminally discernible benefits of moderate levels of activity in this preparation but, by contrast, patterned intense high-frequency stimulation (100 Hz) *ex vivo* accelerated synaptic degeneration. Thus, the sensorimotor differences in protection conferred by Wld^S^ protein expression cannot be explained by differences in the ongoing endogenous activity of their severed distal axons. Moreover, we also found that preconditioning axons, either with chronic disuse or by voluntary exercise, also rendered motor nerve terminals more sensitive to the effects of axotomy (Brown et al. 2015).

Intensive activity was shown many years ago, in the context of a different and natural degenerative phenomenon, to accelerate the withdrawal of synapses that occurs during developmental synapse elimination (O’Brien et al. 1978; Thompson, 1983). Similar effects are suggested indirectly by some experiments on the effects of electrical stimulation or stressful activity on the onset and progression of disease signs in a mouse model of amyotrophic lateral sclerosis (ALS; Lepore et al. 2010; Alvarez et al. 2013). These observations further support conjecture about the mechanistic similarities between synaptic remodeling in development, synaptic degeneration in models of neurodegenerative disease, and axotomy-induced Wallerian degeneration (Gillingwater & Ribchester, 2003; Conforti et al. 2014).

## Age need not weary us …

It will be interesting to find out whether insights into the gene-dose dependence age-sensitivity, and apparent activity-dependence of the protection by *Wld*^*S*^ might be obtained utilizing the advantages of *ex vivo* preparations as well as other animal models, such as *Drosophila*, which lend themselves to analysis using powerful molecular genetic, anatomical, electrophysiological and optical techniques (Eaton et al. 2002; Peled & Isacoff, 2011; Avery et al. 2012; Osterloh et al. 2012; Melom et al. 2013; Ribchester et al. 2013; Ford & Davis, 2014; Peled et al. 2014; Robinson et al. 2014). These preparations also provide opportunities for screening the efficacy and benefits of potentially neuroprotective compounds (Di Stefano et al. 2015) or investigating the mechanism of action of environmental toxins, including pesticides (Dissanayake et al. 2012).

Ageing is the most important risk factor for several neurodegenerative diseases, including Alzheimer’s disease and ALS; and several mechanistic similarities and therapeutic targets to the end stages of axotomy-induced, Wallerian degeneration have been identified. This is perhaps not surprising in light of the view that these diseases are likely to be primary axonopathies (Conforti et al. 2014). But, as the *modus operandi* for investigation of mechanosensory and neuromuscular structure and function, epitomized historically by the Durham School in general and by Bob Banks’ research in particular attests, nature is full of surprises (Bewick et al. 2005; Simon et al. 2010; Shenton et al. 2014), and astonishing natural phenomena of both structure and function will no doubt continue to inform and delight neurobiologists for many years beyond Bob’s well-earned retirement.

## Conflict of Interest

The author has no conflicts of interest to declare.
